# Autoencoder-Based Unsupervised Surface Defect Detection Using Two-Stage Training

**DOI:** 10.3390/jimaging10050111

**Published:** 2024-05-05

**Authors:** Tesfaye Getachew Shiferaw, Li Yao

**Affiliations:** 1 School of Computer Science and Engineering, Southeast University, Nanjing 211189, China; tesfaye@seu.edu.cn; 2 Key Laboratory of Computer Network and Information Integration, Southeast University, Ministry of Education, Nanjing 211189, China

**Keywords:** autoencoder, surface defect detection, structural similarity, perceptual similarity, artificial defect generation

## Abstract

Accurately detecting defects while reconstructing a high-quality normal background in surface defect detection using unsupervised methods remains a significant challenge. This study proposes an unsupervised method that effectively addresses this challenge by achieving both accurate defect detection and a high-quality normal background reconstruction without noise. We propose an adaptive weighted structural similarity (AW-SSIM) loss for focused feature learning. AW-SSIM improves structural similarity (SSIM) loss by assigning different weights to its sub-functions of luminance, contrast, and structure based on their relative importance for a specific training sample. Moreover, it dynamically adjusts the Gaussian window’s standard deviation (σ) during loss calculation to balance noise reduction and detail preservation. An artificial defect generation algorithm (ADGA) is proposed to generate an artificial defect closely resembling real ones. We use a two-stage training strategy. In the first stage, the model trains only on normal samples using AW-SSIM loss, allowing it to learn robust representations of normal features. In the second stage of training, the weights obtained from the first stage are used to train the model on both normal and artificially defective training samples. Additionally, the second stage employs a combined learned Perceptual Image Patch Similarity (LPIPS) and AW-SSIM loss. The combined loss helps the model in achieving high-quality normal background reconstruction while maintaining accurate defect detection. Extensive experimental results demonstrate that our proposed method achieves a state-of-the-art defect detection accuracy. The proposed method achieved an average area under the receiver operating characteristic curve (AuROC) of 97.69% on six samples from the MVTec anomaly detection dataset.

## 1. Introduction

In the ever-evolving landscape of industrial automation, surface defect inspection plays a crucial role in ensuring product quality and propelling the journey toward smarter manufacturing. Surface defects are common in many industrial products, such as fabric [1], steel [2], wood [3], and ceramic [4]. By meticulously examining surfaces for imperfections like scratches, cracks, or discoloration, automated systems guarantee consistent product integrity, minimizing the risk of faulty components reaching consumers. This not only enhances brand reputation and customer satisfaction but also prevents costly recalls and rework, streamlining production processes. Moreover, automated defect inspection paves the way for further automation by providing valuable data for process optimization.

In recent decades, various methods have been proposed for surface defect inspection, mainly categorized into traditional and deep learning-based methods. Traditional methods can be further divided into different categories, including texture feature-based methods, color feature-based methods, and shape feature-based methods [5]. Texture feature-based methods analyze the surface texture patterns to identify defects like scratches, cracks, and unevenness [6]. Color feature-based methods utilize color variations to detect defects like stains, discoloration, and foreign objects. Song et al. [7] proposed a classification method based on the percentage color histogram feature and feature vector texture of an image block for wood surface defect detection. Shape feature-based methods extract and analyze geometric features of defects like size, shape, and depth. They often rely on edge detection, blob analysis, or geometric measurements to identify the defect shape. Wang et al. [8] proposed a hybrid method based on the Fourier transform and Hough transform for the detection of surface-cutting defects on magnets. Tsai et al. [9] proposed a method based on global Fourier image reconstruction and template matching for detecting and locating small defects on electronic surfaces. Traditional methods are capable of extracting features like texture, color, and shape from an image surface. However, traditional methods require domain expertise to design relevant features, struggle with complex textures and new defect types, and are sensitive to variations in lighting and imaging conditions. In contrast, deep learning methods have a strong automatic feature extraction ability and good generalization on a large amount of data. Deep learning methods can be categorized as supervised and unsupervised learning.

Many supervised methods have been proposed for the detection and localization of surface defects in the past decade. Ren et al. [10] proposed an automatic surface defect detection using a pre-trained deep learning network. PGA-Net [11] realizes pixel-wise detection using a pyramid feature fusion and global context attention network. Tabernik et al. [12] presented a segmentation-based deep learning architecture for surface defect detection. Many methods [13,14,15] have been proposed for printed circuit board (PCB) defect detection and localization based on YOLO. Cheng et al. [16] proposed a deep neural network DEA_RetinaNet (RetinaNet) with difference channel attention and adaptively spatial feature fusion for steel surface defect detection. However, fully supervised methods [17,18,19] demand carefully annotated bounding boxes within datasets, a process that can be time consuming, laborious, and prone to inconsistencies. Furthermore, these methods often struggle with unseen defects not encountered in the training data, potentially requiring an ever-expanding dataset, making data collection an ongoing challenge.

Unlike supervised learning methods, which require labeled data for training, unsupervised learning approaches operate with unlabeled normal samples. They learn the underlying distribution from these normal samples during training and identify instances that deviate significantly from this learned distribution as potential defects. This capability allows unsupervised methods to detect previously unseen defects [20,21,22]. Schlegl et al. [23] proposed an anomaly detection generative adversarial network (AnoGAN) to learn the distribution of defect-free texture image patches using GAN techniques. It then detects defects by searching for a latent sample that reproduces a given input image patch. Bergmann et al. [24] proposed a defect inspection method by applying a structural similarity to an autoencoder (AE-SSIM). An unsupervised reconstruction-based method for surface defect detection using a combined structural similarity and mean absolute (L1) loss was proposed in [25]. Bionda et al. [26] proposed a deep autoencoder for anomaly detection based on Complex Wavelet Structural Similarity (CW-SSIM). Chamberland et al. [27] proposed a method to detect defects on cast components using a convolution neural network (CNN) autoencoder. While effective at detecting and localizing most defects, these methods face challenges when dealing with defects that closely resemble the normal background and do not achieve a noise-free reconstruction of the normal background.

Surface defect detection methods trained only on normal samples tend to produce higher reconstruction errors for defect areas compared to the normal background. This indicates their ability to identify deviations from the learned normal patterns. Using the residual (difference) between the input and the reconstructed image, we can localize the defects. However, this approach faces two main challenges:

1. Partial defect reconstruction: Sometimes, the trained model might also reconstruct the defective region, thereby diminishing its ability to distinguish between defective and non-defective regions.
2. Noise-free normal background: Even if defects are identified, the reconstructed normal background often contains noise, making it harder to isolate the defects accurately.

Therefore, achieving a clear and informative residual image requires a high-quality normal background reconstruction and non-reconstruction of the defect. Finding this balance between the reconstruction of a high-fidelity normal background and accurate defect detection has been a significant challenge.

In this paper, we propose an autoencoder-based method with training in two stages. The first stage of training is exclusively conducted only on normal samples, and the second stage is training on normal and artificially defective samples using the model weight from the first stage. Experiments in [25] demonstrated that using the mean difference for the luminance calculation in the structural similarity (SSIM) metric [28] can be problematic when used as a loss function for image reconstruction tasks. This is because the mean is insensitive to the range of pixel values within a local area, leading to identical SSIM scores for images with the same average brightness but significantly different light–dark variations. Inspired by this, we propose an adaptive weighted structural similarity (AW-SSIM) loss by introducing addition instead of multiplication between the luminance, contrast, and structure sub-functions. This allows for the removal of the independence between the three sub-functions to compensate for the luminance’s limitations through contrast or structure. Additionally, we dynamically adjust the standard deviation (σ) for the Gaussian window. This parameter governs the spread of the window, influencing the level of detail captured during SSIM calculations.

Artificial anomalies have recently gained widespread use in enhancing a model’s ability to distinguish the defective part from the normal region of an image. AFEAN [29] uses artificial anomaly generation by combining defect-free images and random masks. CutPaste [30] uses a method that involves cropping a rectangular image patch and pasting it at a random location within a larger image. CMA-AE [31] uses a general artificial anomaly generation algorithm and involves cropping a rectangular area from a natural image in the ImageNet dataset [32] and pasting it at a random location within a normal training image. In our work, we propose an artificial defect generation algorithm (ADGA). This algorithm generates artificial defects that closely mimic natural defects by creating defects with varying shapes and sizes.

To enhance the reconstructed image quality and achieve noise-free reconstruction on the normal background image, we introduce a combined structural and perceptual loss function. For the second stage of training, Learned Perceptual Image Patch Similarity (LPIPS) loss [33] is used in combination with the AW-SSIM to enhance the model’s ability to reconstruct a high-quality and noise-free normal background. The LPIPS aligns well with a human’s perception of the image quality. Unlike traditional loss functions that directly compare pixel values between images, the LPIPS operates by extracting features from the images and comparing them in a latent space using a pre-trained model. Our experiment results prove that the introduction of the LPIPS loss in the second stage of training improves the model’s performance significantly in achieving a high-quality normal background reconstruction. In summary, our key contributions are as follows:

1. We propose a two-stage training strategy involving normal training samples and training samples with artificial defects.
2. The AW-SSIM loss function is proposed, removing the independence between the three sub-functions of the SSIM and dynamically adjusting the standard deviation (σ) for the Gaussian window.
3. We propose an artificial defect generation algorithm (ADGA), a novel algorithm specifically designed to create artificial defects that closely resemble various real-world defects.
4. To improve the quality of normal background reconstruction and defect identification, we propose a combined SSIM and LPIPS loss function for the second stage of training.

The remainder of this paper is organized as follows. Section 2 introduces the related works of surface defect detection. In Section 3, the proposed method, including each improvement, is discussed in detail. Section 4 presents a set of experiments that demonstrate the performance of our method. Finally, Section 5 summarizes our work.

## 2. Related Works

In recent years, defect detection methods based on positive training samples without labels have gained significant attention. The primary reason for this lies in the fact that the effectiveness of training deep learning models is primarily influenced by both the quantity of training samples and the quality of annotations. Autoencoders (AEs) [34] and generative adversarial nets [20] along with their variants have been used to train defect detection models only on defect-free training samples. Some of the proposed GAN-based methods are f-AnoGAN [35], OCGAN [22], and GPND [36]. Popular variants of the autoencoder used in defect detection tasks include the standard convolutional autoencoder (CAE) [37], the variational autoencoder (VAE) [21], and the adversarial autoencoder (AAE) [38]. The CAE has the simplest model and training procedure of these three. Furthermore, the CAE outperforms the other two variants in certain cases, as demonstrated in [39].

Unsupervised training with convolutional autoencoders (CAEs) focuses on learning the normal patterns within the data. During training, the autoencoder analyzes normal images and learns to reconstruct them accurately. This means that it captures the underlying patterns and features that represent normal instances in the training samples. When presented with a test image containing an unseen defect, the trained model will likely struggle to accurately reconstruct that specific region. Different CAEs based the unsupervised method for defect detection have been proposed. Yang et al. [40] proposed a multiscale feature clustering-based fully convolutional autoencoder (MS-FCAE) for defect inspection on textured surfaces utilizing multiple CAE subnetworks at different scale levels. Although traditional loss functions like the mean absolute error (L1) and mean squared error (MSE) loss work for training autoencoders, they only focus on pixel-level differences. This neglects the image’s underlying structure, often leading to blurry reconstructions that hinder effective defect detection. To overcome this limitation, methods utilizing structural similarity loss have been introduced. Bergmann et al. [24] introduced a method that adopts SSIM as a loss function for surface defect inspection. Hu et al. [41] introduced a surface defect inspection method based on a reconstruction network using a combined structural and L1 loss. A deep autoencoder using CW-SSIM for detecting anomalous regions in textured images was proposed in [26]. Despite improvements, these methods still face challenges. They struggle to achieve noise-free background reconstructions and often reconstruct defects during testing. Memory-augmented autoencoders [31,42,43] were proposed to solve the partial reconstruction of defects. Memory-augmented autoencoder-based methods depend on restoring defects for inspection and often struggle to restore complex defects. Furthermore, the memory bank used to store the latent representations of training samples introduces additional computational overhead.

Besides autoencoders, generative adversarial networks (GANs) [20] offer another approach for unsupervised defect detection. In this method, the GAN’s generator learns the data distribution of normal images by analyzing a large number of defect-free samples. F-AnoGAN [35] realizes fast GAN-based anomaly detection. GANomaly [44] uses conditional GAN-based anomaly detection using an encoder–decoder–encoder generator framework. Skip-GANomaly [45] introduced a skip-connection to GANomaly to improve the reconstruction quality of the image background. Xiao et al. [46] proposed a memory-augmented adversarial autoencoder (MemAAE) that utilizes a memory mechanism to manipulate latent features. Despite their success in detecting and localizing diverse defects, these GAN-based methods struggle with balancing a noise-free normal background reconstruction and accurate defect localization.

## 3. Methodology

This section delves into the details of our proposed unsupervised defect detection method. Initially, we present the overall network architecture used in our work. Next, we discuss the proposed AW-SSIM, an important component that enhances our model’s ability to capture the most important features during training. Following this, we introduce our artificial defect generation algorithm (ADGA), which plays a pivotal role in training the model to recognize real-world defects effectively. Finally, we discuss how the proposed combined AW-SSIM and LPIPS loss functions improve the model’s ability to reconstruct a noise-free normal background while accurately detecting defects.

### 3.1. Overall Network Architecture

**Figure 1:**
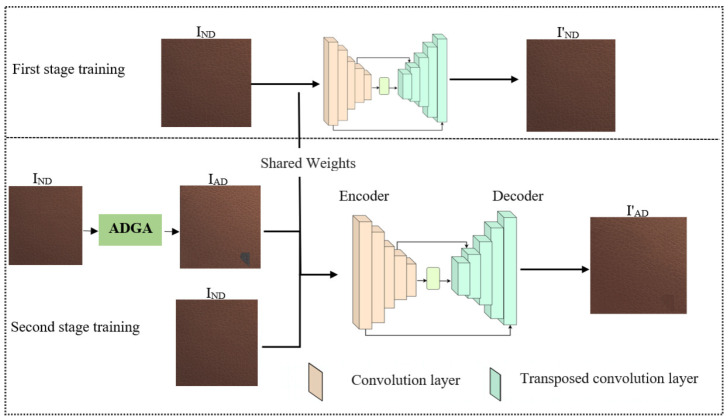
The overall network architecture of our proposed method. In the first stage of training, the model is trained on normal samples ($I_{ND}$) only. This establishes the model’s baseline understanding of what constitutes a normal sample. During the second stage of training, the model weights from the first stage and artificially defective samples ($I_{AD}$) generated by the ADGA in combination with normal samples are used to train the model to detect defects.

The overall architecture of the proposed method is shown in Figure 1. It relies on three key components: the encoder, decoder, and artificial defect generation algorithm (ADGA). The encoder compresses the input image into a lower-dimensional representation using a series of convolutional layers, each employing a sequence of convolution, batch normalization, and activation functions. This compressed representation is then fed into the decoder, which utilizes transposed convolutional layers to progressively upsample the information and reconstruct the original image with high fidelity. Similar to the encoder, each transposed convolutional layer employs a sequence of transposed convolution, batch normalization, and activation functions. The ADGA algorithm create images with artificial defects of various shapes and sizes for the second stage of training. Our model incorporates skip-connections to significantly enhance the performance of the autoencoder. These skip-connections facilitate better information flow between the encoder and decoder, preserving critical fine-grained details in the reconstructed image. While experimenting with various skip-connection configurations, we found that utilizing two strategically placed connections yielded optimal results. Notably, applying skip-connections to all layers resulted in partial defect reconstruction.

The training is performed in two stages. In the first stage, the training is performed only on non-defective training samples ($I_{ND}$), focusing on learning the inherent characteristics of normal images. First, the input image is divided into 128 × 128 patches, and the latent representation is extracted by the encoder. Then, the latent features are fed into the decoder to obtain the reconstructed image (IND′). This stage lays the foundation for the model to understand what constitutes a normal image. Using the weights learned from the first stage of training, the second stage focuses on equipping the model for defect detection. Here, images with artificially introduced defects ($I_{AD}$) created by the ADGA are paired with their non-defect counterparts ($I_{ND}$). During training, these pairs are fed into the model. The normal samples ($I_{ND}$) are used as references to improve the reconstruction performance of the model by comparing a normal sample with the reconstructed image (IAD′) from its artificially defective counterpart ($I_{AD}$). This strategy guides the model to improve its reconstruction abilities while simultaneously learning to identify defects.

### 3.2. Artificial Defect Generation Algorithm (ADGA)

**Figure 2:**
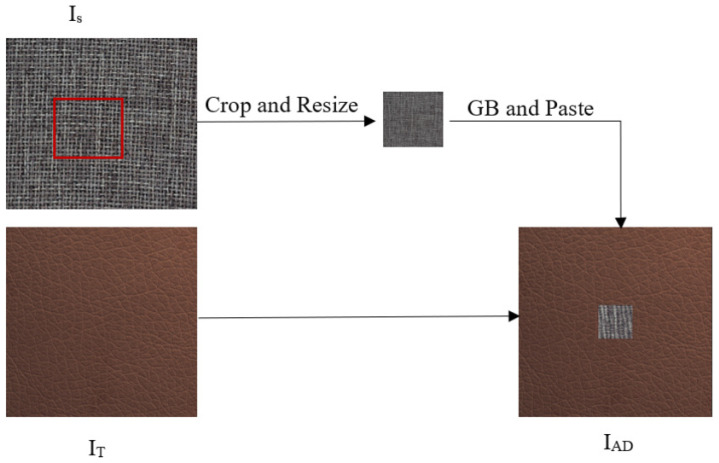
The process of generating artificially defective training samples for the second stage of training. The ADGA cuts a part of a source image ($I_{s}$) from a random position with a random vertex from 3 to 7, applies a Gaussian blur, and pastes it to a random position in the target image ($I_{T}$). The ADGA generates artificially defective training samples with defects of different sizes and shapes.

For the accurate simulation of real-world defect occurrences, artificial defect generation must carefully consider the target image’s ($I_{T}$) specific details where defects will be introduced. Previous methods, such as Cutpaste [30] and CMA-AE [31], generate artificial anomalies by cropping a rectangular shape from a random source image and pasting it at a random position within a target image. Our approach involves cutting a portion from source images with varying shapes and sizes, and then pasting it randomly onto the target image as shown in Figure 2. The source images ($I_{s}$) are carefully selected to represent realistic defects based on the background color and appearance of the target image. We randomly cut a portion of the source image ($I_{s}$) by selecting vertices ranging from 3 to 7, resulting in an irregular shape. Then, the section is resized to a randomly chosen dimension. To smoothly integrate this irregularly shaped portion into the target image ($I_{T}$), we apply a Gaussian blur. This blurring softens the transition at the edges, ensuring a more natural and visually appealing blend with the background.

(1) $$I_{\mathrm{AD}}=\mathrm{Paste}\left(I_{T},\mathrm{GB}\left(\mathrm{Resize}\left(\mathrm{crop}\left(I_{S}\right)\right)\right)\right)$$

**Figure 3:**
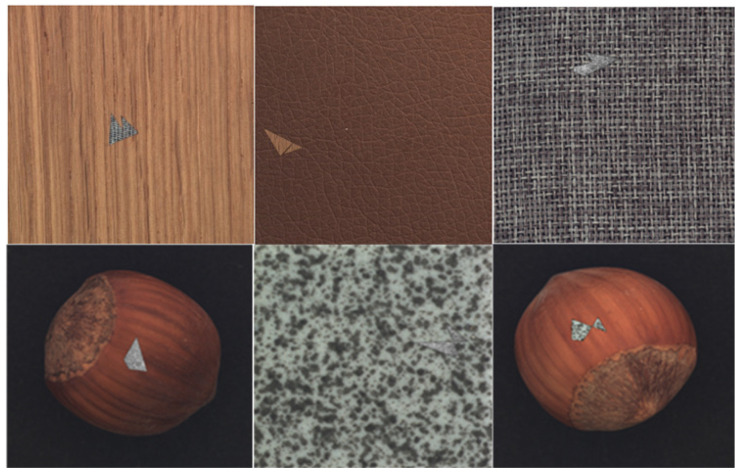
Defects generated by the ADGA on different training samples. The ADGA generates defects that simulate real-world defects with different shapes and sizes. Source images ($I_{s}$) that match the appearance and the color of the normal background in the target images ($I_{T}$) are selected during the defect generation.

Here, $I_{AD}$ is an image with an artificial defect, $I_{T}$ is a normal image that is used as a target image on which to paste the cropped section from the source image ($I_{s}$), and GB is Gaussian blur. The crop () operation represent the cropping of a portion of a source image, Resize () represents the resizing of a cropped portion, and the Paste () operation represents the pasting of the cropped and resized portion to the target image. As shown in Figure 3, the proposed artificial defect generation algorithm (ADGA) can create defective images with defects of different sizes and shapes.

### 3.3. SSIM Loss Function Improvement

SSIM stands out as an image quality assessment metric that aligns closely with human perception, unlike traditional metrics like the mean squared error (MSE) and mean absolute error (MAE). While the MSE and MAE focus only on pixel-wise differences, the SSIM takes into account aspects like luminance, contrast, and structure, mirroring how humans judge image quality. To calculate the structural similarity (SSIM), we compare two images, *x* and *y*, of size m × n. The SSIM assesses similarity based on three key aspects of human perception: luminance, contrast, and structure. These aspects are represented by functions l(x,y), c(x,y), and s(x,y) respectively. Each sub-function’s value is determined based on the mean, variance, and covariance of the corresponding pixel values in the two images using sliding windows of size ξ×ξ with a step size of 1 in both the horizontal and vertical directions.

Luminance is calculated using the mean of the patches from the two images. We obtain a luminance score near 0 if the brightness of the patches differs greatly, and of 1 if they are similar:

(2) $$l\left(x,y\right)=\frac{2\mu_{x}\mu_{y}+C_{1}}{\mu_{x}^{2}+\mu_{y}^{2}+C_{1}}$$

(3) $$C_{1}={\left(K_{1}L\right)}^{2}$$

where $\mu_{x}$ and $\mu_{y}$ are the local mean for images *x* and *y*, respectively, and $C_{1}$ is a constant used to avoid instability when the denominator value is close to zero. $K_{1}$ is a small constant (0.01 recommended), and *L* represents the dynamic range of the pixel values in the images.

We compute the contrast using the variance of the corresponding patches from the two images. The contrast score compares the difference in “texture” between the image patches:

(4) $$c\left(x,y\right)=\frac{2\sigma_{x}\sigma_{y}+C_{2}}{\sigma_{x}^{2}+\sigma_{y}^{2}+C_{2}}$$

where $\sigma_{x}^{2}$ and $\sigma_{y}^{2}$ are the local variances for the corresponding images *x* and *y*. $C_{2}={\left(K_{2}L\right)}^{2}$, and $K_{2}$ is a small constant value (recommended to be 0.03).

Structure is computed using the cross-covariance $\sigma_{xy}$ between the corresponding patches in *x* and *y*. The score is high when both patches contain an edge with the same location and orientation, but low if the two patches disagree on the location of an edge:

(5) $$s\left(x,y\right)=\frac{\sigma_{xy}+C_{3}}{\sigma_{x}\sigma_{y}+C_{3}}$$

where $C_{3}$ is a small constant, usually $C_{3}=\frac{1}{2}C_{2}$.

Finally, we have

(6) $$\mathrm{SSIM}\left(x,y,\xi \right)={\left(l(x,y)\right)}^{\alpha }\cdot {\left(c(x,y)\right)}^{\beta }\cdot {\left(s(x,y)\right)}^{\gamma }$$

where ξ is the sliding window size. We used (11×11) in our work. The parameters α, β, and γ are set to one in this paper.

The SSIM loss calculation uses multiplication between the three sub-functions to bring independence between these factors, as shown in Equation (6). As demonstrated in [25], the mean used to measure the difference in luminance limits the SSIM’s ability to differentiate between an image with high light–dark variance and an image with more consistent light and dark if the two images have the same mean luminance value. To compensate for the mean with the variance and co-variance values, we should remove the independence between the luminance and other sub-functions through using addition instead of multiplication.

(7) AW-SSIM(x,y,ξ)=αl(x,y)+βc(x,y)+γs(x,y)

**Figure 4:**
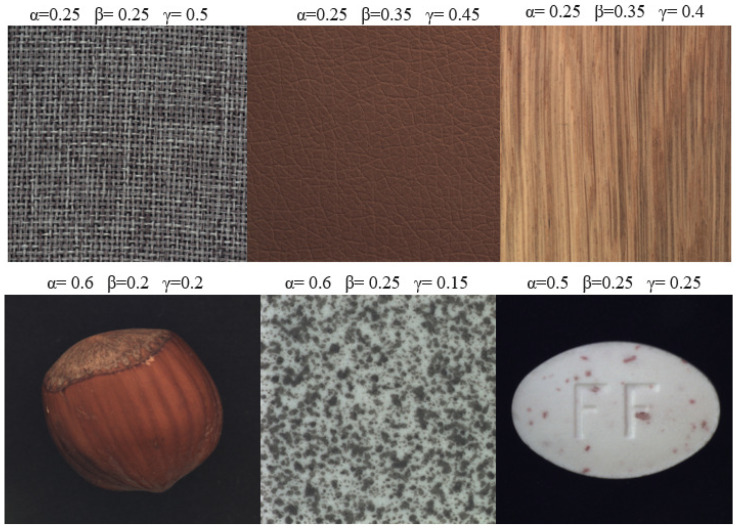
The weights given to each sub-function of the SSIM based on its relative importance for different training samples. For carpet, leather, and wood data, the highest weight is assigned to structure (γ), since the structure is more important than brightness and contrast. The highest weight is assigned to luminance (α) for a training samples like tile, pill, or hazelnut since brightness, lighting, and color changes are more important than structure.

For AW-SSIM, we apply addition between the three sub-functions, as shown in Equation (6). In addition, we multiply each sub-function, luminance, contrast, and structure, by a constant α, β, and γ, respectively. This multiplication with a constant determines the relative importance of each sub-function in the overall AW-SSIM calculation. In weighting the influences of the luminance, contrast, and structure based on the training data content, the model’s robustness and generalization ability are improved. According to our experiment, using different weights based on each sub-function’s importance for a specific training samples, as shown in Figure 4, shows a better result than using equal weights for all sub-functions. The calculations of the luminance, contrast, and structure values in AW-SSIM are the same as in the standard SSIM.

The weighting factors in the AW-SSIM equation (α, β, and γ) allow us to prioritize different aspects of image similarity. Assigning a higher weight (α) to the luminance component (l(x,y)) emphasizes the importance of overall brightness differences. Conversely, a larger weight (β) for the contrast component (c(x,y)) prioritizes preserving local variations in intensity, which are crucial for images containing intricate textures or fine details. Finally, the structural similarity component (s(x,y)) with weight (γ) captures how well the underlying structures, like edges and patterns, are preserved between the images. A higher weight for the structure would prioritize preserving the spatial arrangement of features, which is crucial for maintaining the perceptual quality of the image.

We tested this on different samples from the MVTec AD dataset [47] and observed a better result than using equal weights for each sub-function. For example, if we take the carpet sample from the MVTec AD dataset, assigning the structure sub-function a higher weight yields a better result since the intricate details of the weave, such as the thickness of the threads and the way they interlace, are what define the visual texture of the carpet image, as shown in Figure 4. The SSIM’s structure sub-function is specifically designed to capture these details. For training samples like tile, hazelnut, and pill, a higher weight is given to luminance, since luminance is more important than the other sub-functions in capturing the lighting variations and changes across the images. The weight values for each sub-function are selected after extensive experimentation to determine how they affect the overall performance of the trained model on specific training samples.

The SSIM calculation assesses the structural similarity between two images by comparing three key features: luminance, contrast, and structure. This comparison is performed on localized image regions called patches. To incorporate spatial information, a Gaussian window function is applied to each corresponding image patch. This window typically acts as a sliding window, moving pixel by pixel across both patches. The Gaussian window plays a crucial role in emphasizing the central region of each patch. Pixels closer to the center receive higher weights during the SSIM calculation. This weighting scheme gradually decreases toward the edges of the window, diminishing the influence of outlying pixels. The calculation of the Gaussian window is performed using the selected window size and the standard deviation at a specific position in an image patch, as shown in Equation (8):

(8) $$G\left(n\right)=e^{-\left(\frac{{\left(n-\xi \right)}^{2}}{2\sigma^{2}}\right)}$$

where G(n) represents the value of the Gaussian window at position *n*, and ξ is the sliding window size. We used a window size of 11 × 11 in our work. σ is the standard deviation for the Gaussian window. The above formula creates a one-dimensional window, and then we can create a two-dimensional window using the normalized one-dimensional window. The Gaussian window assigns weights to pixels based on their distance from the center of the window. Higher weights are assigned to points closer to the center.

**Figure 5:**
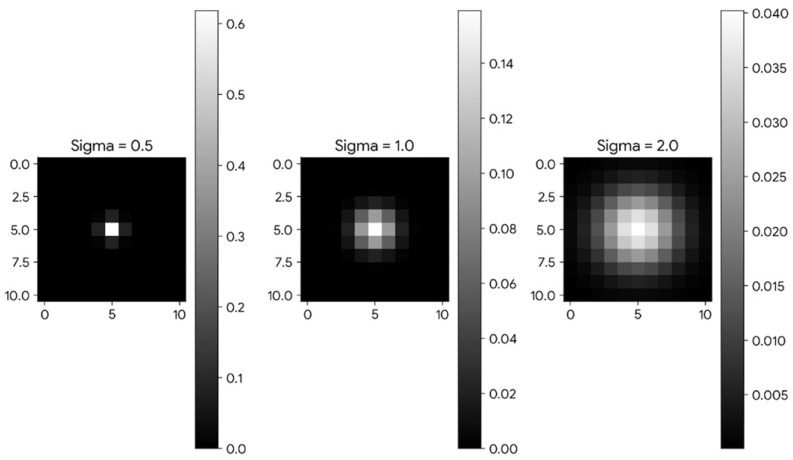
The effects of different values of standard deviation (σ) for the Gaussian window during SSIM loss calculation. A small standard deviation (σ) value for the Gaussian window will assign pixels heavier weights at the center of the window. A bigger σ value results in a wider window covering more distant pixels.

The standard deviation σ of the Gaussian window determines how weights are assigned to pixels within the sliding window. A smaller σ creates a narrower window, concentrating weight near the center and reducing the influence of distant pixels, as shown in Figure 5. Conversely, a larger σ results in a wider window, spreading weights more evenly and increasing the influence of distant pixels.

However, using a single σ value for the entire Gaussian window is not optimal. A small σ amplifies noise, while a large σ blurs fine details. To address this, we propose a dynamic approach. Initially, σ is set to a small value. During training, it is gradually incremented by a small constant after processing every $P_{H}\times P_{W}$ patch of the training images. Importantly, the training samples are shuffled after each epoch, and σ is reset to its initial value before being incremented again. This approach ensures that different window sizes are applied to different patches within each image throughout training. By dynamically adjusting σ, we can reduce the negative effect of using a fixed σ value, leading to better noise control and improving the preservation of fine details in the final results.

(9) $$\sigma =\sigma +\left\lceil \frac{\mathrm{number}\;\mathrm{of}\;\mathrm{samples}}{\mathrm{batch}\;\mathrm{size}}\right\rceil \times \frac{H}{P_{H}}\times \frac{W}{P_{W}}\times k$$

Here, σ is the standard deviation after each epoch, which depends on the number of training samples and the sizes of the patches ($P_{H}\times P_{W}$) in patch-based training. H and W are the height and width of the input image, respectively, and *k* is a small constant added to σ after each patch of size $P_{H}\times P_{W}$ in the input image.

### 3.4. Combined AW-SSIM and LPIPS Loss for Stage Two of Training

Structural loss like the SSIM loss is a type of metric aimed at measuring low-level pixel-wise similarity. In contrast, perceptual loss focuses on capturing high-level visual similarity as perceived by humans. Perceptual loss uses pre-trained deep neural networks inspired by the human visual system. In a perceptual loss function, we compare high-level features extracted from pre-trained convolutional neural networks (CNNs). The goal is to generate visually pleasing images by minimizing perceptual differences between them. These differences, such as content and style discrepancies, may not be apparent at the pixel level. Unlike traditional pixel-wise loss functions, which directly compare raw pixel values, perceptual loss functions leverage feature maps from various layers of a pre-trained network. These networks are typically pre-trained on large datasets like ImageNet [32]. By extracting these feature maps from both the target image and the reconstructed image, we can compute the differences in the high-level features that the network has learned to detect, such as edges, textures, and patterns.

In this study, we used LPIPS loss [33], a perceptual loss that utilizes deep features extracted from pre-trained networks to assess the perceptual similarity between two images. In our case, we chose AlexNet [48], which is a smaller and faster architecture compared to other pre-trained networks. During the calculation of the LPIPS loss, two patches are first passed through a series of convolutional layers of the pre-trained network to extract high-level features. The perceptual similarity between the two patches is estimated by comparing their feature vectors extracted from a pre-trained network using a distance metric. The LPIPS metric is calculated as the average distance between the feature vectors of the two patches across all layers of the network.

In the second stage of training, we use a combined LPIPS and AW-SSIM loss, with a higher weight assigned to the AW-SSIM loss. The AW-SSIM loss evaluates local structural similarities between images, effectively identifying pixel-level variations. Meanwhile, the LPIPS concentrates on perceptual similarity, spotlighting visually noticeable abnormalities even with minor pixel-wise differences, making it valuable for detecting subtle texture changes or shape inconsistencies. This combined loss approach facilitates the high-quality reconstruction of the normal background while effectively identifying defects.

(10) $$L\left(I_{\mathrm{ND}},I{’}_{\mathrm{AD}}\right)=\alpha \cdot \mathrm{AW}\text{-}\mathrm{SSIM}\left(I_{\mathrm{ND}},I{’}_{\mathrm{AD}}\right)+\left(1-\alpha \right)\cdot \mathrm{LPIPS}\;\mathrm{loss}\left(I_{\mathrm{ND}},I{’}_{\mathrm{AD}}\right)$$

Here, L(IND,IAD′) is the combined structural and perceptual loss, $I_{ND}$ is the original image without defects, and IAD′ is the reconstructed image from the artificially defective image ($I_{AD}$).

## 4. Experimentation

In this study, we thoroughly evaluated the proposed method’s performance based on a series of experiments. We compared its overall effectiveness against that of previous state-of-the-art methods on different samples on the MVTec AD dataset [47]. Additionally, ablation experiments were conducted to delve into the influence of each improvement implemented in our approach.

For our experiments, we used diverse anomaly detection samples sourced from the MVTec AD dataset [47], including leather, carpet, hazelnut, pill, wood, and tile textures. In total, 1660 and 714 samples were used for training and testing, respectively. All images were resized to 512 × 512 pixels during training. Training was performed using an Adam optimizer trained for a total of 25 epochs in two stages. To quantitatively assess the performances of various methods, we chose the area under the receiver operating characteristic curve (AuROC) as the evaluation metric. This metric is insensitive to threshold variations and provides a more accurate evaluation of the models’ inspection capabilities.

### 4.1. Overall Performance Comparison

To validate the effectiveness of our proposed method, we compared its defect detection performance against those of several prominent anomaly and defect detection methods, including AE-SSIM [24], AnoGAN [23], f-AnoGAN [35], MS-FCAE [40], MemAE [42], TrustMAE [43], VAE [49], ACDN [50], AFEAN [29], and CMA-AE [31]. Table 1 summarizes the quantitative analysis results. Our method achieved a superior performance across various sample types, including wood, carpet, hazelnut, and pill. Additionally, it obtained the second-best results on the tile and leather samples.

**Figure 6:**
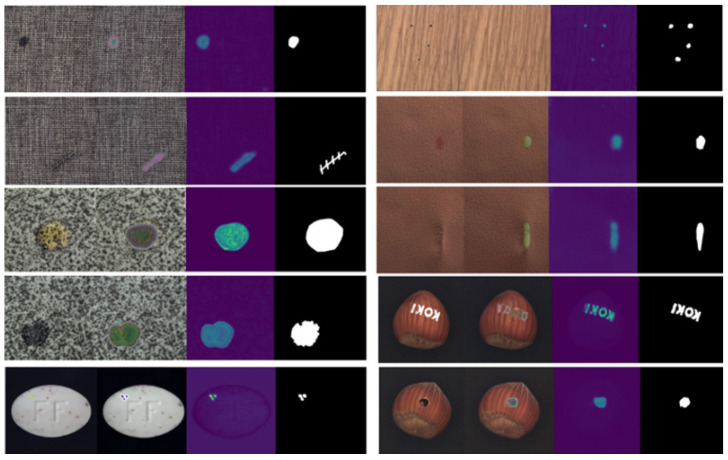
The defect inspection results for our method on different samples from the MVTec AD dataset [47]. From **left** to **right** are the defective input images, the reconstructed images, residual images, and the ground truth. The model was able to reconstruct the normal region of the testing images efficiently and struggled to reconstruct the defective regions; the residual between the original and the reconstructed image can be used for locating the defects.

Figure 6 presents the inspection results of our proposed method on six different samples. We tested the proposed method on different samples with different kinds of defects like color stains, holes, contamination, etc. By employing a two-stage training process, using AW-SSIM as a loss function, utilizing the ADGA, and combining structural and perceptual losses, the model achieves a clear reconstruction of the normal background. The trained model is unable to reconstruct defective regions. By taking the residual between the input and the reconstructed image, we can localize the defects accurately.

### 4.2. Ablation Study

**Figure 7:**
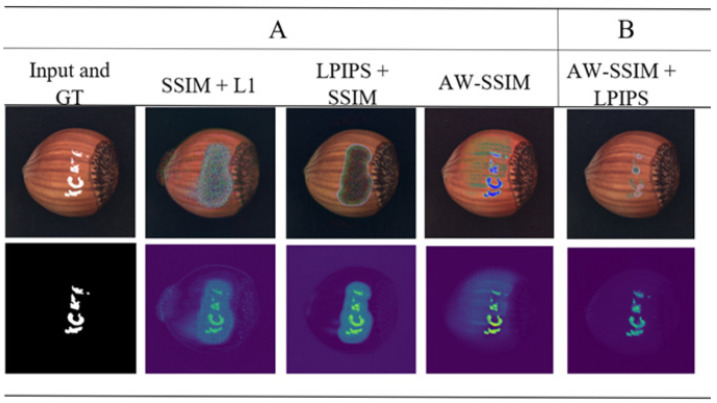
The test results of the ablation experiment: (**A**) one-stage training and (**B**) two-stage training. The first row of images are the input and reconstructed images. The second row has the ground truth and residual images. For each combined loss, a higher weight was assigned to the SSIM and AW-SSIM loss.

To validate the individual contributions of each proposed improvement in our method, we conducted a series of ablation experiments. For fair comparisons, all evaluated improvements employed the same parameter settings. The qualitative and quantitative results of these comparisons are presented in Figure 7 and Table 2, respectively.

#### 4.2.1. The Influence of AW-SSIM

We improved the SSIM loss function to guide the model toward learning essential features from training samples. This was achieved by assigning weights to the three sub-functions (luminance, contrast, and structure) based on their relative significance. This weighting scheme guides the model to prioritize the most crucial features. Furthermore, we dynamically adjusted the Gaussian window’s standard deviation (σ) during the SSIM calculation. This optimizes the balance between using small and large σ values. In Figure 7, the second column shows the model’s performance trained with the unmodified SSIM loss, and the result shows a poor reconstruction in the normal region surrounding the defect. Conversely, the AW-SSIM loss performs better in noise reduction and defect localization, as shown in the fourth column. Moreover, Table 2 reveals a 4.16% improvement in the AUROC.

#### 4.2.2. The Influence of the Combined LPIPS and AW-SSIM Loss

We proposed a combined LPIPS and AW-SSIM loss to achieve both high-quality, noise-free normal background reconstruction and accurate defect detection. While the performance of the AW-SSIM is good in defect localization, its background reconstruction suffers from noise and quality issues. Interestingly, the combined LPIPS and AW-SSIM loss achieve a better normal background reconstruction compared to AW-SSIM loss, as shown in Figure 7’s third column. However, the combined LPIPS and AW-SSIM loss is not able to localize the defect accurately in only one-stage training. While the LPIPS loss introduces a little computational overhead, it significantly boosts the model performance in accurately localizing defects and high-quality normal background reconstruction, as shown in Figure 7.

#### 4.2.3. Influence of Two-Stage Training

During one-stage training, the model trained with the AW-SSIM loss is able to effectively localize defects but struggles to achieve a high-quality, noise-free reconstruction of the normal region of the image. Conversely, using the combined LPIPS and AW-SSIM loss gives a high-quality normal background reconstruction, but the defect localization performance is poor. To overcome these limitations, we propose a two-stage training approach that leverages the strengths of both methods. In the first stage, the model trains only on normal samples using the AW-SSIM loss function, effectively learning the background characteristics. These learned weights are then transferred to the second stage, which utilizes artificially generated defects created by the ADGA and employs a combined LPIPS and AW-SSIM loss. This two-stage approach allows the model to accurately detect defects while maintaining a high-quality background reconstruction compared to with only one-stage training. As shown in the last column of Figure 7, this strategy successfully achieves both goals. Additionally, Table 2 shows that the two-stage training method achieves the highest AUROC score compared to single-stage training. Another significant advantage of using LPIPS loss and applying a two-stage training is a reduced training time compared to that in the single-stage approach with a traditional loss function like the MSE. This efficiency gain comes from two factors: firstly, LPIPS leverages a pre-trained model for loss calculation, and secondly, the second stage of training benefits from the weights pre-trained in the first stage, significantly reducing the overall training time.

## 5. Conclusions

This paper proposes an unsupervised learning method for surface defect detection. It directly addresses the challenge of acquiring labeled training data using positive samples and samples with artificially generated defects, circumventing the need for real defective images within the training dataset. This approach is particularly significant, as collecting and labeling real defective data can be a difficult and time-consuming process.

The proposed method employs a two-stage training strategy. The first stage of training is performed only on normal samples, utilizing the AW-SSIM loss function. AW-SSIM loss prioritizes the most important features during training by assigning different weights to the three sub-functions (luminance, contrast, and structure). Additionally, it achieves a balance between effective noise control and the preservation of fine details by dynamically adapting the Gaussian window’s standard deviation (σ). In the second stage of training, artificially defective samples and normal samples are used to train the model and enhance its ability to localize defects. The proposed artificial defect generation algorithm (ADGA) generates artificial defects that closely mimic real-world defects. Furthermore, the second stage of training uses a combined loss function incorporating both AW-SSIM and LPIPS loss, aiming to improve the quality of normal background reconstruction.

Extensive experiments demonstrate the proposed method’s ability to detect defects while achieving the high-quality reconstruction of normal backgrounds. Through an ablation study, we rigorously evaluated the effectiveness of each proposed improvement within our method. In future research, we will focus on improving LPIPS loss based on the requirement of training samples, improving the ADGA to generate more complex and realistic defects, and using a pre-trained network as a feature extractor.

## Figures and Tables

**Table 1:** Different methods’ performances evaluated based on the AuROC on six samples from the MVTec AD dataset [47]. The proposed method outperformed different state-of-the-art methods on various samples and achieved the best average result.

Category	AE-SSIM	AnoGAN	f-AnoGAN	MS-FCAE	MemAE	TrustMAE	VAE	ACDN	AFEAN	CMA-AE	Ours
Tile	59.00	50.00	72.00	53.20	70.76	82.48	65.40	93.60	85.70	**98.82**	96.56
Wood	73.00	62.00	74.00	81.20	85.44	92.62	83.80	92.90	92.20	96.96	**97.10**
Leather	78.00	64.00	83.00	91.70	92.91	98.05	92.50	98.40	96.10	**99.13**	98.76
Carpet	87.00	54.00	66.00	78.20	81.16	98.53	73.50	91.10	91.40	91.25	**99.20**
Hazelnut	96.60	87.00	63.15	78.50	81.16	97.15	98.80	94.10	92.80	97.10	**98.89**
Pill	89.50	93.25	64.07	80.60	77.88	89.90	93.50	92.80	89.60	92.65	**95.64**
average	80.51	68.38	70.37	77.23	81.55	93.12	84.50	93.81	91.3	95.98	**97.69**

Bold text indicates the best results and underlined text indicates the second-best results.

**Table 2:** A quantitative comparison of the proposed improvements in our method. The improved SSIM loss (AW-SSIM), LPIPS loss, and the proposed two-stage training improved the AuROC significantly.

Training	One-Stage Training			Two-Stage Training
Loss	SSIM + L1	LPIPS + SSIM	AW-SSIM	AW-SSIM + LPIPS
AuROC	86.7	90.86	95.60	98.89

## Data Availability

The dataset used in this study is open access and can be found here: https://www.mvtec.com/company/research/datasets/mvtec-ad, accessed on 18 March 2024.
